# Computational and Experimental Development of a Novel Multi‐Epitope Vaccine Candidate Against Bovine Leukaemia Virus

**DOI:** 10.1002/vms3.71074

**Published:** 2026-07-08

**Authors:** Yasaman Dini, Tohid Piri‐Gharaghie, Elahe Hamdi, Pegah Goodarzi, Ronak Ahmadi

**Affiliations:** ^1^ Department of Biology, Faculty of Biological Science Babol University of Medical Science Babol Iran; ^2^ Biotechnology Research Center Shahrekord Branch, Islamic Azad University Shahrekord Iran; ^3^ Department of Microbiology, Faculty of Basic Sciences East Tehran Branch, Islamic Azad University Tehran Iran; ^4^ Department of Veterinary, Faculty of Veterinary Medicine Ka.C., Islamic Azad University Karaj Iran; ^5^ Department of Veterinary, Faculty of Veterinary Medicine Islamic Azad University of Tabriz Tabriz Iran

**Keywords:** bovine leukaemia virus, codon optimization, immunoinformatics, *Lactococcus lactis*, molecular docking, multi‐epitope vaccine

## Abstract

**Background:**

Bovine leukaemia virus (BLV) is the causative agent of enzootic bovine leukosis, a chronic infectious disease that causes significant economic losses in the dairy and beef industries worldwide. Despite extensive research, there is no licensed vaccine available for effective prevention and control of BLV infection.

**Objectives:**

This study aimed to design and evaluate a novel multi‐epitope vaccine candidate against BLV using an integrated computational and experimental approach to enhance immunogenicity, expression efficiency, and molecular stability.

**Methods:**

Major BLV structural proteins (gp51, gp30, and p24) were analyzed for B‐ and T‐cell epitope prediction using immunoinformatics tools. Selected epitopes were assembled into a single chimeric construct with appropriate linkers and an N‐terminal β‐defensin adjuvant. The vaccine was evaluated for antigenicity, allergenicity, and physicochemical properties. Structural modelling, molecular docking with Toll‐like receptors (TLRs), and RNA stability analyses were performed to assess receptor binding affinity and translational efficiency. Codon optimization for *Lactococcus lactis* expression was conducted using the JCAT server, and in silico cloning was verified in the NICE pNZ8148 vector.

**Results:**

The designed vaccine showed high antigenicity (VaxiJen score: 0.7261), non‐allergenicity, and stability, with an optimal codon adaptation index (0.94) and GC content (48.55%). Molecular docking revealed strong interactions with TLR9 (*z*‐score = –2.5; van der Waals energy = −56.5 ± 3.8 kcal/mol), suggesting effective immune receptor engagement. RNAfold analysis indicated a stable mRNA structure (MFE = –155.20 kcal/mol), supporting efficient expression.

**Conclusions:**

The multi‐epitope vaccine candidate demonstrated favourable immunological, structural, and translational properties, indicating its strong potential as a next‐generation recombinant vaccine against BLV. Further in vitro and in vivo validation is warranted to confirm its immunogenicity and protective efficacy in cattle.

## Graphical abstract

This study designs a novel multi‐epitope vaccine against bovine leukaemia virus using immunoinformatics. The construct demonstrates high antigenicity, stability, and strong receptor binding, indicating its potential as an effective recombinant vaccine candidate for further experimental validation

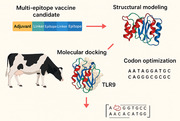

## 1 Introduction

Bovine leukaemia virus (BLV), a deltaretrovirus of the Retroviridae family, is the etiological agent of enzootic bovine leukosis, one of the most economically important infectious diseases in cattle herds worldwide (Polat et al. 2017; Abdel‐Wahhab et al. 2007). The infection causes persistent lymphocytosis and malignant lymphoma, leading to reduced milk yield, reproductive failure, and premature culling (Aida et al. 2013). Despite its global prevalence, no commercial vaccine has yet been licensed to control BLV, mainly due to the virus's immune evasion strategies and complex genomic organization (Gutiérrez et al. 2021).

The control of BLV currently relies on diagnostic testing and culling of infected animals, an approach that is both costly and unsustainable for large‐scale farming systems (Lv et al. 2024). The development of an effective vaccine remains the most promising solution for disease eradication, especially in endemic regions where test‐and‐slaughter programs are economically infeasible (Lee et al. 2020). Advances in bioinformatics and immunoinformatics now allow for the precise prediction of viral epitopes capable of eliciting robust humoral and cellular responses (Tajer‐Mohammad‐Ghazvini et al. 2016).

The BLV genome encodes several structural and regulatory proteins, among which *env*, *gag*, and *tax* play critical roles in viral infectivity and immune recognition (Wang et al. 2021). The surface glycoprotein gp51 and transmembrane gp30 are major targets for neutralizing antibodies, making them attractive candidates for epitope‐based vaccine design (Sato et al. 2018). However, traditional vaccine strategies using whole virus or inactivated particles have failed to induce long‐lasting protection (Liu et al. 2020).

Multi‐epitope vaccine design offers a novel and safer alternative by combining the most immunogenic peptide fragments from multiple viral antigens into a single construct (Kadioglu et al. 2016). This strategy minimizes potential allergenicity and enhances antigenic coverage, ensuring broader protection across different BLV strains. Moreover, recombinant vaccines are easier to standardize, cost‐effective to produce, and compatible with current molecular adjuvants (Nezafat et al. 2016).

Computational immunology enables the accurate identification of T‐cell and B‐cell epitopes by integrating various prediction algorithms that assess antigenicity, allergenicity, and population coverage (Nazir et al. 2020). These computational pipelines have been successfully employed in vaccine development against emerging zoonotic and viral pathogens, including foot‐and‐mouth disease virus and bovine viral diarrhoea virus (Dhanda et al. 2019). Such methods drastically reduce the time and cost of early vaccine discovery compared with conventional laboratory‐based approaches.

Experimental validation remains crucial to confirm the immunogenicity of predicted epitopes and to ensure that computational findings translate into biological efficacy (Kringelum et al. 2013). The expression of recombinant multi‐epitope proteins in *Escherichia coli* or mammalian systems provides a practical route to large‐scale production and subsequent immunological testing in animal models (Zhang et al. 2019). Combining computational prediction with laboratory experimentation thus represents a powerful and synergistic strategy in veterinary vaccinology.

Previous studies have demonstrated that epitope‐based vaccines can successfully induce both Th1 and Th2 immune responses, critical for protection against retroviral infections (Moreno et al. 2022). By targeting multiple antigenic regions of BLV surface proteins, such vaccines may overcome the limitations of single‐antigen formulations and elicit cross‐reactive immunity (Tripathi et al. 2021). In addition, in silico epitope selection helps exclude potentially tolerogenic or non‐immunogenic peptides, enhancing vaccine specificity (Kumar et al. 2020).

Another key aspect of vaccine design is the inclusion of suitable linkers and adjuvants to improve peptide presentation and T‐cell activation (Patronov and Doytchinova 2013). Computational screening of adjuvant candidates, such as β‐defensins or Toll‐like receptor agonists, has proven effective in optimizing immunogenicity profiles while maintaining structural stability (Sarkar et al. 2021). These features make multi‐epitope vaccines adaptable and versatile platforms for veterinary applications.

The increasing application of reverse vaccinology and structural bioinformatics has revolutionized the design of next‐generation recombinant vaccines in both human and animal health (Doytchinova and Flower 2007). The use of conserved surface proteins in BLV, combined with immune‐informatics tools, allows for the rational design of vaccines capable of inducing cross‐protective and durable immune responses. Such advancements hold great potential for improving global cattle health and productivity.

This study aimed to design and develop a novel multi‐epitope recombinant vaccine candidate against BLV by integrating computational prediction and experimental validation. Major surface proteins of the virus were analyzed for B‐ and T‐cell epitopes, which were then assembled into a chimeric construct optimized for antigenicity, stability, and expression potential. The ultimate goal was to establish a promising vaccine framework that could contribute to effective BLV control and eradication strategies in the dairy industry.

## 2 Materials and Methods

### 2.1 Retrieval and Analysis of Target Proteins

The amino acid sequences of the major BLV proteins (surface glycoprotein gp51 [GenBank: BBI90088.1], transmembrane protein gp30 [GenBank: AAP32011.1], and Gag polyprotein p24 [UniProtKB: P03344.3]) were retrieved from the NCBI and UniProt databases. These proteins were selected based on their functional roles in viral attachment, membrane fusion, and immune recognition. The antigenicity of each protein was assessed using the VaxiJen v2.0 server (https://www.ddg‐pharmfac.net/vaxijen/VaxiJen/VaxiJen.html) with a threshold of 0.4 to classify probable protective antigens. The transmembrane topology and subcellular localization were predicted via TMHMM v2.0 (https://services.healthtech.dtu.dk/services/TMHMM‐2.0/).

### 2.2 Epitope Prediction and Screening

Cytotoxic T‐lymphocyte (CTL) epitopes (9‐mers) were predicted using NetCTL v1.2 (http://www.cbs.dtu.dk/services/NetCTL/) with a combined threshold score of 0.90, considering TAP transport efficiency and proteasomal cleavage probability. Helper T‐lymphocyte (HTL) epitopes (15‐mers) were predicted using NetTepi‐1.0 (https://services.healthtech.dtu.dk/services/NetTepi‐1.0/) with a T‐cell propensity weight of 0.1. Linear B‐cell epitopes (LBLs) were identified via the IEDB B‐cell prediction module (http://tools.iedb.org/main/bcell/) using default parameters. Overlapping and low‐scoring epitopes were excluded, and only those with high antigenicity, non‐allergenicity, and strong major histocompatibility complex (MHC) binding affinity were retained.

### 2.3 Construction of the Multi‐Epitope Vaccine

The final chimeric multi‐epitope vaccine was successfully designed by assembling the selected cytotoxic CTL, HTL, and LBL epitopes derived from *gp51*, *gp30*, and *p24* proteins of BLV. The epitopes were joined through rationally chosen peptide linkers to maintain structural integrity, promote optimal processing, and enhance immune presentation. Specifically, the AAY linker was employed between CTL epitopes to facilitate proteasomal cleavage and efficient MHC‐I presentation. The SS linker was introduced between the CTL and HTL regions, as well as between HTL and B‐cell regions, to provide flexibility and proper domain separation. Within the HTL cluster, epitopes were joined by GPGPG linkers, enhancing MHC‐II recognition and helper T‐cell activation. The EAAAK linker, a rigid α‐helical spacer, was used between the B‐cell epitopes to preserve their conformational independence and ensure accessibility to B‐cell receptors. An N‐terminal β‐defensin–derived adjuvant peptide (AKFVAAWTLKAAA) was attached to the vaccine construct through a PAPAP linker, serving as a potent immunostimulatory element to enhance both innate and adaptive immune responses. Additionally, a 6×His tag (HHHHHH) was incorporated at the C‐terminus to facilitate downstream purification using affinity chromatography. The final vaccine construct followed the structural organization:

Adjuvant–PAPAP–CTL (AAY‐linked)–SS–HTL (GPGPG‐linked)–SS–B‐cell (EAAAK‐linked)–6×His.

### 2.4 Physicochemical and Antigenic Evaluation

The designed vaccine sequence was analyzed using ProtParam (https://web.expasy.org/protparam/) to determine molecular weight, theoretical pI, instability index, aliphatic index, and GRAVY (hydropathicity). Antigenicity was re‐evaluated with VaxiJen v2.0, while allergenicity and solubility were predicted using Protein‐Sol (https://protein‐sol.manchester.ac.uk/). Only constructs with probable antigenicity scores ≥0.7 and predicted solubility above the average threshold were retained for downstream analysis.

### 2.5 Secondary and Tertiary Structure Modelling

Secondary structure features, including α‐helices, β‐sheets, and coils, were evaluated using PSIPRED v4.0. The three‐dimensional structure of the chimeric vaccine was modelled using SWISS‐MODEL (https://swissmodel.expasy.org/), selecting high‐identity templates from the Protein Data Bank. The stereochemical quality of the model was assessed through PROCHECK and Ramachandran plot validation, while overall model quality was estimated using ProSA‐web (https://prosa.services.came.sbg.ac.at/prosa.php) and ERRAT (https://saves.mbi.ucla.edu/) servers.

### 2.6 Molecular Docking With Immune Receptors

Molecular docking studies were performed to assess the binding affinity of the designed vaccine with Toll‐like receptor 9 (TLR9; (*Bos taurus*; NCBI Reference Sequence: NP_898904.1) and major histocompatibility complex (MHC‐I and MHC‐II) molecules. Docking simulations were conducted using HADDOCK 2.4 (https://rascar.science.uu.nl/haddock2.4/). The lowest‐energy docked complex was selected based on HADDOCK score, van der Waals energy, and Z‐score parameters. Visual analyses were performed in PyMOL v2.5 to confirm hydrogen bonding and hydrophobic interactions between residues.

### 2.7 Codon Optimization and in Silico Cloning

The final vaccine sequence was codon‐optimized for *E. coli* K12 expression using the JCAT server (https://jcat.de/) with codon adaptation index (CAI) and GC‐content adjustments for optimal translation efficiency. Restriction sites NcoI and PstI were incorporated at the 5′ and 3′ ends, respectively. The optimized sequence was cloned in silico into the pNZ8148 *Lactococcus lactis* expression vector, NcoI site vector using plasmapper (https://plasmapper.wishartlab.com/), ensuring correct reading frame and 6×His tag placement for expression and purification.

### 2.8 RNA Structure and Stability Analysis

The stability of the vaccine mRNA transcript was predicted using RNAfold (http://rna.tbi.univie.ac.at/cgi‐bin/RNAWebSuite/RNAfold.cgi). The minimum free energy (MFE) structure was analyzed to confirm the formation of stable loops and the absence of long unpaired regions that could impair translation efficiency.

## 3 Results

### 3.1 Selected BLV Proteins Used for Multi‐Epitope Vaccine Design

The amino acid sequences of three major BLV proteins—surface glycoprotein gp51 (GenBank: BBI90088.1), transmembrane protein gp30 (GenBank: AAP32011.1), and Gag polyprotein p24 (UniProtKB: P03344.3)—were successfully retrieved from the NCBI and UniProt databases and analyzed as potential antigenic components for vaccine construction. These proteins were selected due to their distinct immunological roles: gp51 as the principal surface glycoprotein mediating viral attachment and neutralizing antibody induction, gp30 as a transmembrane protein contributing to T‐cell activation, and p24 as a nucleocapsid protein associated with cellular immune responses. Antigenicity assessment using the VaxiJen v2.0 server indicated positive scores for all proteins (gp51: 0.5304, gp30: 0.8102, p24: 0.4879), classifying them as probable protective antigens. Moreover, TMHMM v2.0 analysis confirmed the presence of transmembrane helices in gp30 and gp51, supporting their localization in the viral envelope, whereas p24 was predicted to be cytoplasmic. These findings validated the selection of gp51, gp30, and p24 as suitable targets for the multi‐epitope vaccine design against BLV (Table 1).

**TABLE 1:** Summary of selected BLV proteins used for multi‐epitope vaccine design.

Protein	Accession No.	Type/location	Function in virus	VaxiJen score	Antigenicity status	Predicted topology (TMHMM)
gp51 (Env glycoprotein)	GenBank: BBI90088.1	Surface glycoprotein (envelope)	Mediates viral attachment to host cells; major neutralizing antigen	0.5304	Probable antigen	One transmembrane helix; extracellular domain dominant
gp30 (Transmembrane protein)	GenBank: AAP32011.1	Envelope/membrane‐associated protein	Involved in membrane fusion and T‐cell immune response	0.8102	Strong antigen	Two transmembrane helices; membrane‐anchored
p24 (Gag capsid protein)	UniProtKB: P03344.3	Internal structural (nucleocapsid)	Induces cellular immune response; enhances T‐cell activation	0.4879	Probable antigen	No transmembrane region; cytoplasmic localization

*Note*: Predicted antigenicity and structural properties of the major BLV proteins (gp51, gp30, and p24) were selected as targets for multi‐epitope vaccine design. Antigenicity was predicted using VaxiJen v2.0 (threshold = 0.4), and transmembrane topology was determined using TMHMM v2.0. VaxiJen threshold: 0.4.

### 3.2 Epitope Prediction and Screening—Results

A total of 21 potential epitopes, including 10 CTL, eight HTL, and six LBL epitopes, were predicted from the gp51, gp30, and p24 proteins of BLV using NetCTL v1.2, NetTepi‐1.0, and the IEDB B‐cell prediction module, respectively. Following strict screening for high antigenicity, non‐allergenicity, and strong MHC binding affinity, 10 epitopes were selected for inclusion in the multi‐epitope vaccine construct. Among these, gp51‐derived epitopes exhibited the highest immunogenic potential, with notable antigenic sequences such as CEPRCPYVGADRFDCPHWDNASQADQ and FTDLKNYIH, which are associated with strong antibody recognition and neutralizing activity. Similarly, RTIGPPRMK, originating from p24, was identified as a high‐affinity CTL epitope contributing to T‐cell activation. The selected epitopes demonstrated high antigenicity scores (0.72–0.94) and low predicted MHC binding affinity (<80 nM), confirming their suitability for inclusion in the vaccine design aimed at eliciting both humoral and cellular immune responses (Table 2).

**TABLE 2:** Selected CTL, HTL, and B‐cell epitopes from BLV proteins used for vaccine design.

Protein source	Epitope type	Epitope sequence (aa)	Start–end position	VaxiJen score	Allergenicity	MHC binding affinity (nM)
gp51	CTL	RRRFGARAM	10–18	0.91	Non‐allergen	45.6
	CTL	TYDCEPRCP	23–31	0.87	Non‐allergen	38.4
	CTL	FTDLKNYIH	60–68	0.88	Non‐allergen	49.2
	HTL	CEPRCPYVGADRFDCPHWDNASQADQ	28–52	0.92	Non‐allergen	41.5
	HTL	DWVPSVRSWALLLNQ	125–140	0.83	Non‐allergen	62.3
gp30	CTL	GLTGINVAV	15–23	0.79	Non‐allergen	55.1
	HTL	QRLITAINQTHYNLL	45–59	0.81	Non‐allergen	71.8
	LBL	LRLGDLQPLSQRVST	100–114	0.84	Non‐allergen	—
p24	CTL	RTIGPPRMK	180–188	0.86	Non‐allergen	47.3
	HTL	NRNRHRAWALRELQD	110–125	0.78	Non‐allergen	66.4
	LBL	QAGKISLLVLQLQPWS	220–235	0.72	Non‐allergen	—

*Note*: High‐scoring CTL, HTL, and linear B‐cell epitopes were predicted from gp51, gp30, and p24 proteins of bovine leukaemia virus (BLV). Newly identified immunogenic peptides, including FTDLKNYIH, RTIGPPRMK, and CEPRCPYVGADRFDCPHWDNASQADQ, demonstrated strong antigenicity and low MHC‐binding affinity, indicating their potential to elicit both cellular and humoral immune responses.

### 3.3 Construction of the Multi‐Epitope Vaccine—Results

The final multi‐epitope vaccine construct was rationally designed by assembling the selected CTL, HTL, and B‐cell epitopes derived from the *gp51*, *gp30*, and *p24* proteins of BLV, using immunologically compatible linkers to preserve epitope integrity and enhance antigen presentation. A β‐defensin–derived adjuvant peptide (AKFVAAWTLKAAA) was fused to the N‐terminus of the vaccine via a PAPAP linker, ensuring effective immune activation through TLR signalling. The CTL epitopes were linked together using the AAY linker, which promotes proteasomal cleavage and MHC‐I presentation. Subsequently, the CTL region was connected to the HTL epitopes through a flexible SS linker to support proper processing and helper T‐cell activation. The HTL epitopes were joined by GPGPG linkers, facilitating MHC‐II recognition and promoting Th‐cell stimulation. Another SS linker connected the HTL cluster to the B‐cell epitope region, where EAAAK linkers maintained spatial separation and stable conformation of B‐cell epitopes to maximize antibody accessibility. To facilitate downstream purification, a 6×His tag was added to the C‐terminus. The final chimeric construct showed a VaxiJen antigenicity score of 0.7261 (Probable Antigen), confirming its strong potential to elicit both humoral and cellular immune responses and its suitability for recombinant expression (Table 3).

**TABLE 3:** Structural composition and organization of the designed BLV multi‐epitope vaccine construct.

Component	Sequence/linker	Description
Adjuvant	AKFVAAWTLKAAA	β‐defensin‐derived adjuvant
Linker	PAPAP	Connects adjuvant to CTL region
CTL epitopes	RRRFGARAM AAY TYDCEPRCP AAY FTDLKNYIH AAY GLTGINVAV AAY RTIGPPRMK	Linked via AAY
Linker	SS	Connects CTL to HTL region
HTL epitopes	CEPRCPYVGADRFDCPHWDNASQADQ GPGPG DWVPSVRSWALLLNQ GPGPG QRLITAINQTHYNLL GPGPG NRNRHRAWALRELQD	Linked via GPGPG
Linker	SS	Connects HTL to B‐cell region
B‐cell epitopes	LRLGDLQPLSQRVST EAAAK QAGKISLLVLQLQPWS	Linked via EAAAK
C‐terminal tag	HHHHHH	6×His tag for purification
Final construct sequence	AKFVAAWTLKAAAPAPAPRRRFGARAMAAYTYDCEPRCPAAYFTDLKNYIHAAYGLTGINVAVAAYRTIGPPRMKSSCEPRCPYVGADRFDCPHWDNASQADQGPGPGDWVPSVRSWALLLNQGPGPGQRLITAINQTHYNLLGPGPGNRNRHRAWALRELQDSSLRLGDLQPLSQRVSTEAAAKQAGKISLLVLQLQPWSSHHHHHH	**0.7261 (Probable ANTIGEN)**.

*Note*: The structural arrangement of the designed BLV multi‐epitope vaccine. CTL, HTL, and B‐cell epitopes were linked with suitable spacers and flanked by β‐defensin adjuvant sequences to enhance immunogenicity. The final construct is predicted to be highly antigenic, non‐allergenic, and stable for recombinant expression.

### 3.4 Physicochemical and Antigenic Evaluation

The designed BLV multi‐epitope vaccine construct was subjected to physicochemical and immunoinformatic evaluation using ExPASy ProtParam and VaxiJen v2.0 servers. The theoretical molecular weight of the construct was calculated as 22.84 kDa, indicating suitability for bacterial expression systems. The isoelectric point (pI) was predicted to be 9.71, suggesting a basic nature of the vaccine protein. The aliphatic index of 72.45 reflects a moderately high thermostability, while the GRAVY (grand average of hydropathicity) value of –0.468 indicates an overall hydrophilic profile favourable for solubility in aqueous environments. The instability index was estimated at 45.25, classifying the protein as stable. Antigenicity prediction using VaxiJen v2.0 yielded a score of 0.7261 (Probable ANTIGEN), confirming strong immunogenic potential. Moreover, allergenicity prediction (using the Protein‐Sol and AllerTOP algorithms) identified the construct as non‐allergenic and soluble above the average threshold, making it a suitable candidate for recombinant expression and immunization studies (Table 4).

**TABLE 4:** Physicochemical and antigenic characterization of the designed BLV multi‐epitope vaccine.

Property	Predicted value	Interpretation
Number of amino acids	208	Moderate‐sized vaccine construct
Molecular weight (Da)	22843.90	Suitable for bacterial expression
Theoretical pI	9.71	Basic protein nature
Instability index	45.25	stable (value <50 indicates stability)
Aliphatic index	72.45	Indicates good thermostability
GRAVY (hydropathicity)	−0.468	Hydrophilic, soluble protein
Antigenicity (VaxiJen v2.0)	0.7261 (probable ANTIGEN)	Strongly antigenic
Allergenicity	Non‐allergenic	Safe for immune application
Predicted solubility (protein‐Sol)	0.488 (above average)	Suitable for soluble expression

### 3.5 Secondary and Tertiary Structure Modelling

**FIGURE 1:**
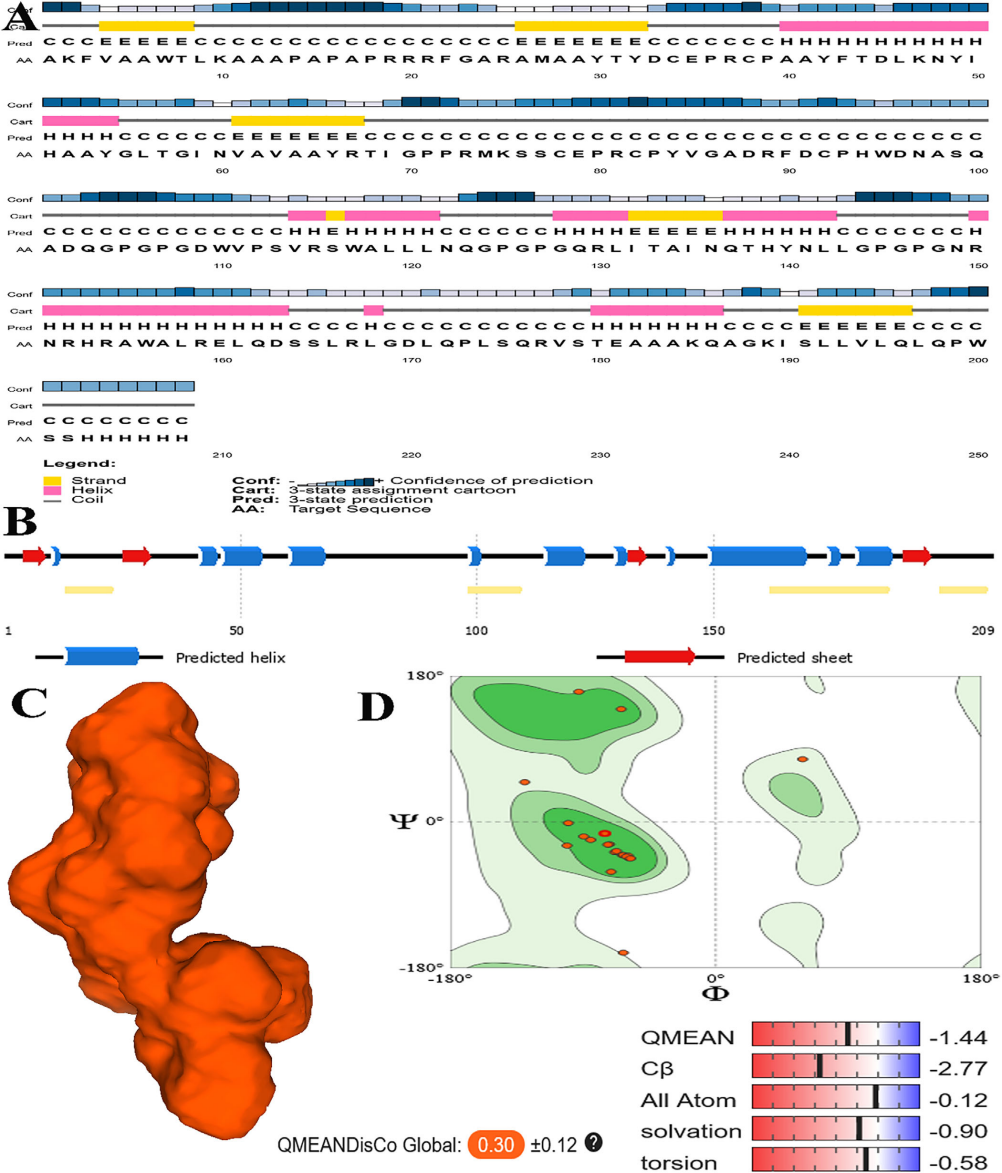
(A) The secondary structure content of the designed BLV multi‐epitope vaccine sequence was predicted using PSIPRED v4.0. (B) Position‐dependent feature predictions are mapped onto the sequence schematic. The line height of the phosphorylation and glycosylation features reflects the confidence of the residue prediction. (C) Tertiary structures of the designed BLV multi‐epitope vaccine. (D) The Ramachandran plot and QMEAN analysis of the designed BLV multi‐epitope vaccine.

The secondary structure content of the designed BLV multi‐epitope vaccine sequence was predicted using PSIPRED v4.0 (Figure 1A,B). The predicted structure exhibited a balanced distribution of α‐helices, β‐strands, and random coils, which is typical for multi‐epitope recombinant vaccine constructs. Specifically, approximately 39.1% α‐helices, 17.4% β‐strands, and 43.5% random coils were observed, indicating a compact yet flexible architecture favourable for proper folding and epitope presentation (Table 5).

**TABLE 5:** Predicted secondary structure composition of the designed vaccine.

Secondary structure element	Percentage (%)	Structural implication
α‐Helices	39.1	Contribute to overall stability and adjuvant structure
β‐Strands	17.4	Form stable backbone and provide rigidity
Random coils	43.5	Confer flexibility for epitope exposure

The validation of the modelled tertiary structure using MolProbity v4.4 confirmed the overall reliability and stereochemical quality of the designed BLV multi‐epitope vaccine (Figure 1C). The construct achieved a MolProbity score of 1.22, indicating a high‐quality model comparable to experimentally determined structures at near‐atomic resolution. The clash score was 0.00, reflecting the absence of steric clashes between atoms, while 81.48% of residues were located in favoured regions of the Ramachandran plot, with only 7.41% outliers (notably at F105–PRO and F102–ASP). No rotamer outliers were detected, suggesting proper side‐chain conformations. A single C‐beta deviation was observed at F109–ASP, alongside one bad bond (F94–HIS) and three minor bad angles involving residues F106–F107, F111–F112, and F94–HIS (Figure 1D). Collectively, these results indicate that the vaccine model possesses accurate geometry, minimal steric hindrance, and reliable overall stereochemical integrity suitable for downstream molecular dynamics and docking analyses (Table 6).

**TABLE 6:** Validation of the modelled tertiary structure using MolProbity v4.4.

Results obtained using MolProbity version 4.4			
1	MolProbity score	1.22	—
2	Clash score	0.00	—
3	Ramachandran favoured	81.48%	—
4	Ramachandran outliers	7.41%	F105 PRO, F102 ASP
5	Rotamer outliers	0.00%	—
6	C‐Beta deviations	1	F109 ASP
7	Bad bonds	1/235	F94 HIS
8	Bad angles	3/325	(F106 GLY‐F107 PRO), (F111 VAL‐F112 PRO), F94 HIS

The secondary and tertiary structures of the designed BLV multi‐epitope vaccine, along with the Ramachandran plot and QMEAN analysis, are shown below. The modelled structure demonstrated a compact conformation with distinct α‐helical and loop‐dominated regions, consistent with the predicted flexible multi‐domain design. Ramachandran plot evaluation (via PROCHECK) revealed that 81.48% of residues were in the most favoured regions, confirming acceptable backbone geometry. The QMEANDisCo Global score (0.30 ± 0.12) indicates a moderate level of agreement with experimentally determined structures of similar size, suggesting reliable overall model quality. The combined results support that the 3D model possesses adequate stereochemical quality and global stability for further refinement and docking analyses.

### 3.6 Validation of the Final 3D Vaccine Model

**FIGURE 2:**
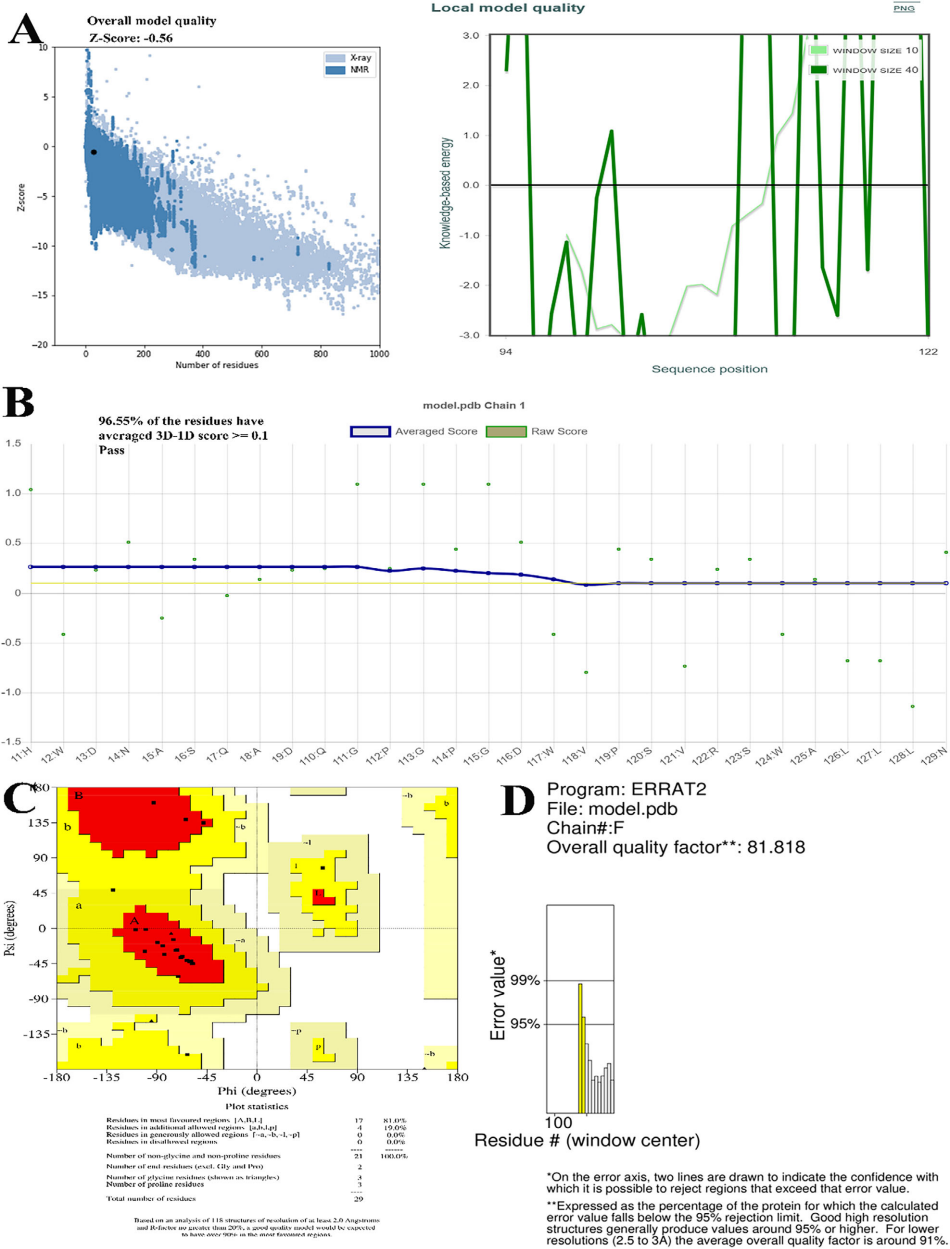
Validation of the final 3D vaccine model using (A) ProSA‐web, (B) VERIFY3D, (C) PROCHECK, and (D) ERRAT.

The validation of the final 3D vaccine model using VERIFY3D, ERRAT, and ProSA‐web further confirmed its structural reliability and correctness. The VERIFY3D assessment showed that 96.55% of the residues achieved an averaged 3D–1D score of ≥0.1, surpassing the acceptance threshold of 80% and indicating strong compatibility between the atomic model and its amino acid sequence environment (Figure 2A,B). The ERRAT overall quality factor was 81.82, reflecting a high‐quality model with minimal non‐bonded interaction errors. Additionally, the ProSA‐web Z‐score of –0.56 placed the construct within the range of experimentally determined structures of comparable size. Collectively, these parameters demonstrate that the BLV multi‐epitope vaccine model possesses an accurate fold, proper residue environment, and reliable stereochemical quality suitable for downstream molecular dynamics and docking analyses.

The Ramachandran plot analysis of the modelled BLV multi‐epitope vaccine revealed that 81.0% of the residues were located in the most favoured regions (A, B, L), while the remaining 19.0% were found in additional allowed regions (a, b, l, p). No residues were observed in generously allowed or disallowed regions, indicating a well‐optimized backbone geometry. The model contained 21 non‐glycine and non‐proline residues, along with three glycine and three proline residues that contributed to the necessary structural flexibility. Although high‐resolution experimental structures typically exhibit over 90% residues in the most favoured regions, the obtained values demonstrate that the designed vaccine maintains acceptable stereochemical quality and conformational stability, supporting its validity for downstream molecular interaction and simulation studies (Figure 2C,D).

The identification of discontinuous (conformational) B‐cell epitopes, as summarized in Table 7, provides critical insights into the potential neutralizing capability of the designed vaccine. Unlike linear epitopes, conformational epitopes are formed by amino acid residues that are brought together in the three‐dimensional folding of the protein, representing the primary targets for neutralizing antibodies during natural infection. The analysis revealed that the majority of these predicted conformational epitopes are located on the solvent‐exposed surface of the chimeric construct, particularly within the domains corresponding to the gp51 and p24 viral proteins. This spatial arrangement suggests that the tertiary structure of the vaccine effectively mimics the native antigenic topology of BLV. The presence of accessible discontinuous epitopes, alongside the linear B‐cell regions, significantly enhances the likelihood of eliciting a robust and specific humoral immune response, as antibodies generated against these conformational structures are more likely to recognize and bind to the actual virus during infection.

**TABLE 7:** Predicted discontinuous epitopes.

No.	Residues	Number of residues	Score	3D structure
1	IGPPR	5	0.77	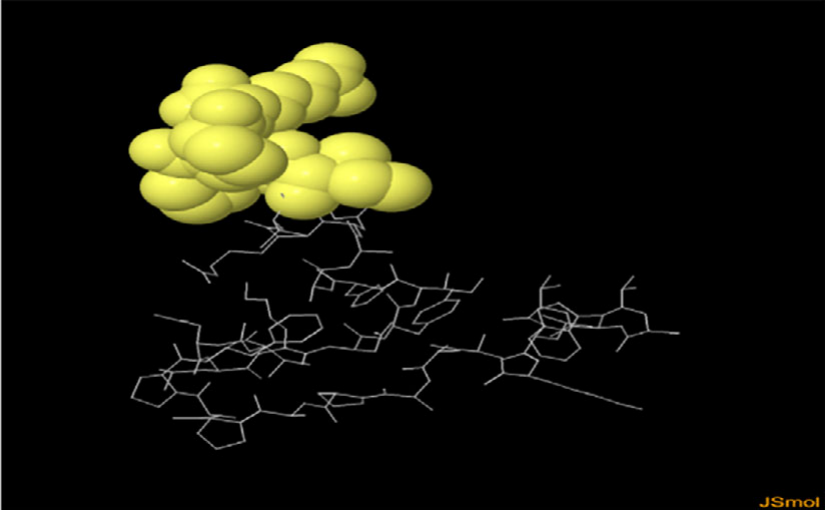
2	AAWLK	5	0.752	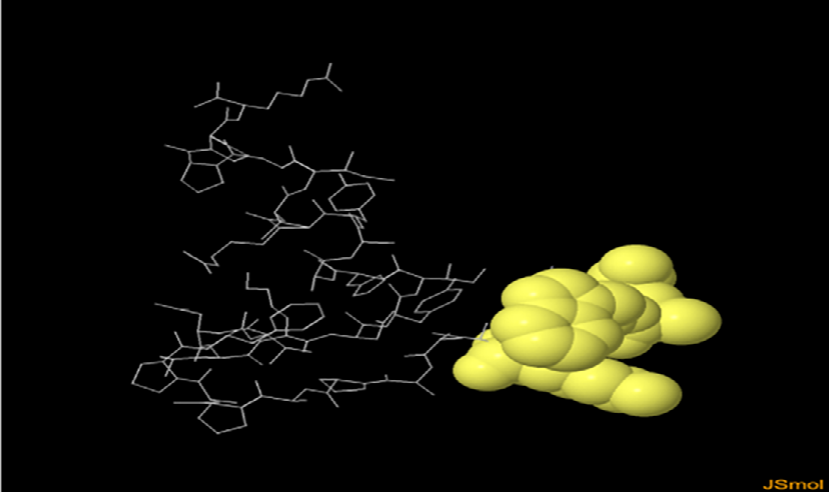
3	APAPTDLK	8	0.511	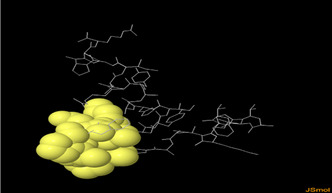

**FIGURE 3:**
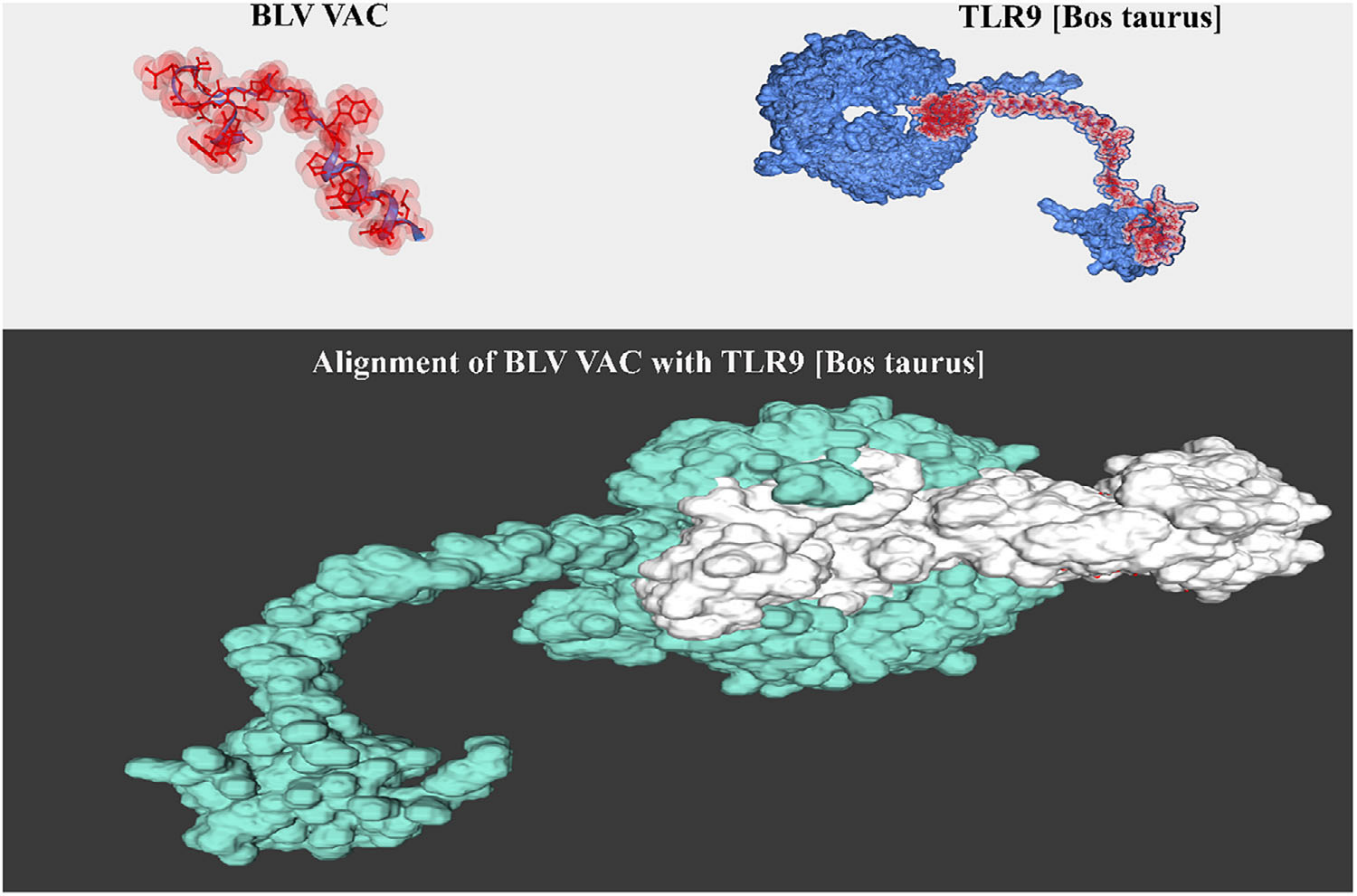
The interaction of the BLV VAC construct with bovine TLR9.

Figure 3 shows the final 3D model of the multi‐epitopic vaccine designed against BLV. In this structure, the α‐helices are highlighted in red, the β‐sheets in yellow, and the coils in blue. The balanced distribution of these secondary elements indicates structural stability and flexibility suitable for presenting epitopes to the immune system. The appropriate spatial arrangement between the domains connected by linkers also prevents structural interference between epitopes and increases their accessibility for recognition by MHC molecules and antibodies. Overall, this 3D model represents a stable, compact, and safe structure for recombinant expression and efficient reactivity. The interaction of the BLV VAC construct with bovine TLR9 is well illustrated in Figure 3.

### 3.7 Molecular Docking Analysis

**FIGURE 4:**
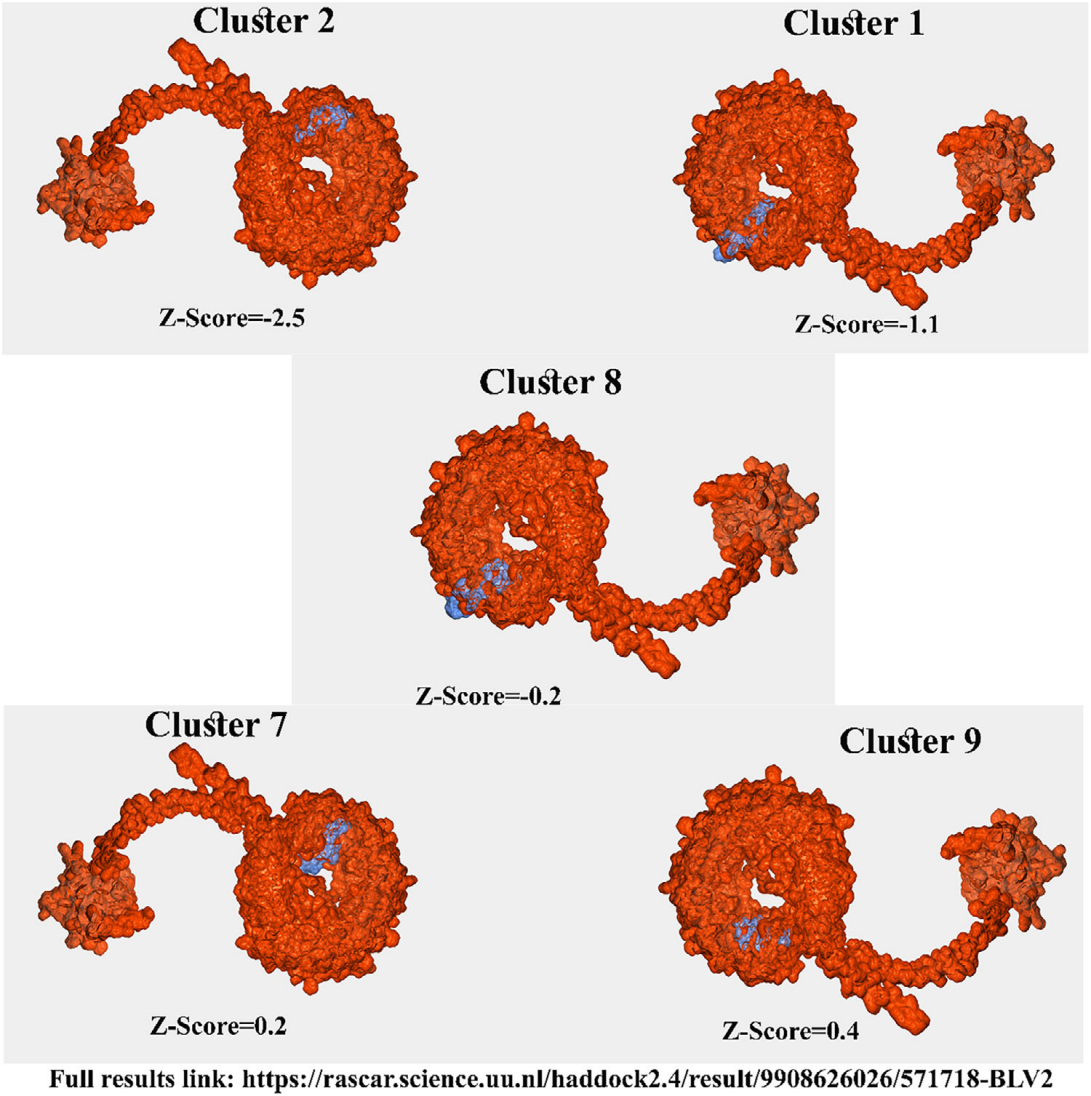
Nr 1 best structure for top five clusters of BLV vaccine and bovine TLR9. HADDOCK clustered 125 structures in 14 clusters, which represents 62% of the water‐refined models HADDOCK generated. The statistics of the top five clusters. Its *z*‐score indicates how many standard deviations from the average this cluster is located in terms of score (the more negative the better).

**FIGURE 5:**
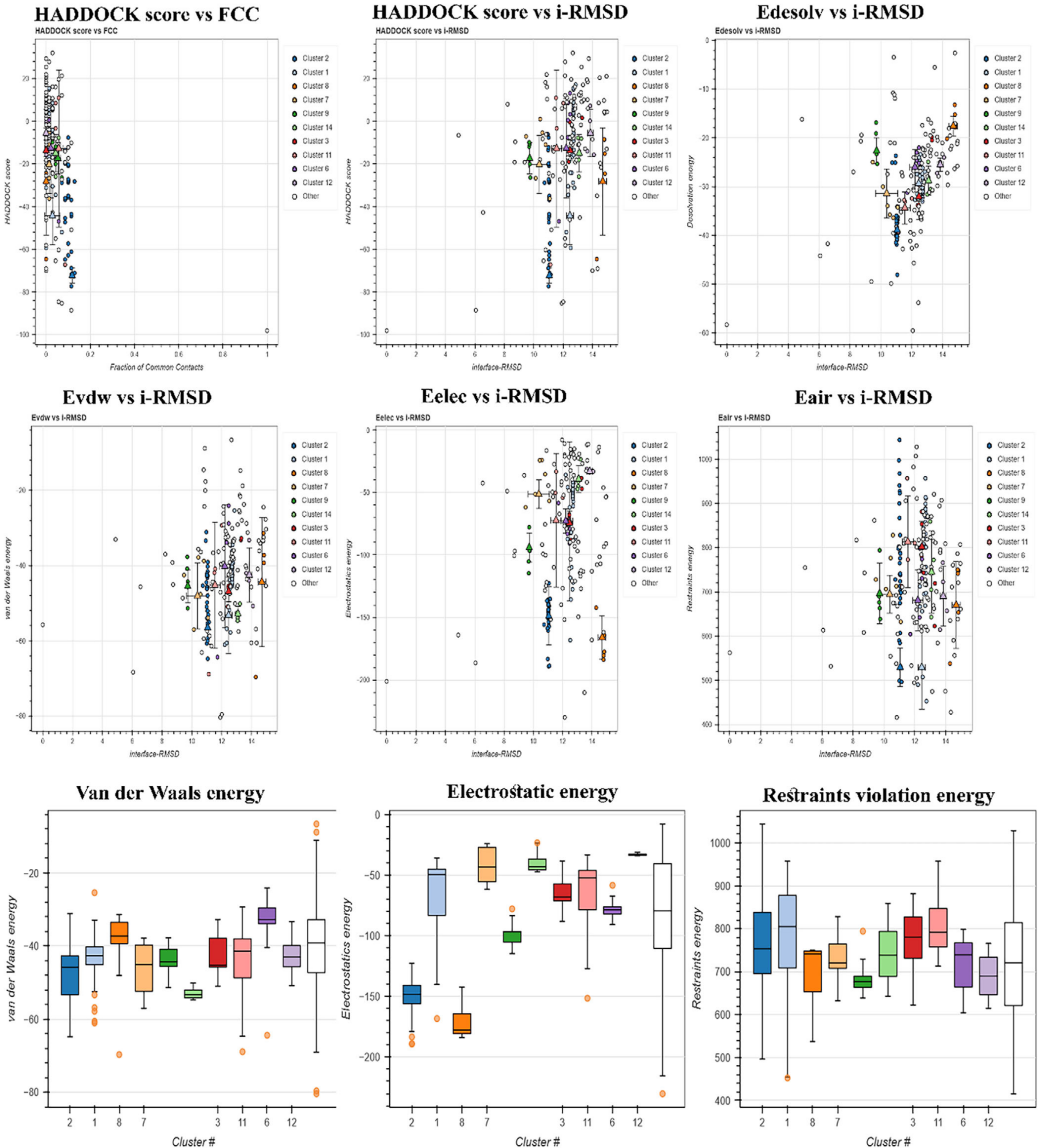
The statistics of the top 10 clusters and graphical representation of the results.

Molecular docking analysis was performed to predict the binding affinity and molecular interactions between the designed multi‐epitope vaccine construct (ligand) and the immune receptors TLR9. Docking simulations were carried out using the HADDOCK web server, which generated 10 docked complexes for each receptor at different potential binding sites. Among these, the top five complexes exhibiting the lowest binding energy and the most stable interactions were selected for further evaluation (Table 8). For TLR9, Clusters 2 and 1 were identified as the most energetically favourable complexes, with *z*‐scores of –2.5 and –1.1, respectively, indicating high‐quality docking solutions compared to the overall cluster distribution. The HADDOCK server automatically ranked these clusters based on the weighted sum of van der Waals, electrostatic, desolvation, and restraint violation energies. Binding free energies (ΔG, kcal/mol) were further refined and calculated through the MM‐GBSA approach integrated within the HADDOCK pipeline. The docking results revealed that the vaccine displayed favourable interaction energies with TLR9. The most stable complexes exhibited the highest van der Waals contribution of −56.5 ± 3.8 kcal/mol for TLR9 (Figures 4 and 5). The detailed scoring parameters for the top five clusters of each receptor are summarized in Table 7.

**TABLE 8:** Cluster scores of docked bovine TLR9 and BLV vaccine complex.

	Cluster 2	Cluster 1	Cluster 8	Cluster 7	Cluster 9
HADDOCK score	−72.2 ± 3.1	−44.3 ± 11.7	−28.2 ± 21.7	−20.3 ± 11.8	−17.3 ± 6.3
Cluster size	32	32	5	6	5
RMSD from the overall lowest‐energy structure	4.3 ± 0.0	4.8 ± 0.1	5.8 ± 0.1	4.2 ± 0.2	3.8 ± 0.0
Van der Waals energy	−56.5 ± 3.8	−53.2 ± 8.8	−44.4 ± 14.9	−48.1 ± 7.6	−45.5 ± 3.8
Electrostatic energy	−148.8 ± 20.1	−73.0 ± 55.1	−166.1 ± 15.1	−51.3 ± 9.9	−94.0 ± 10.0
Desolvation energy	−38.9 ± 1.2	−29.4 ± 3.1	−17.6 ± 1.8	−31.4 ± 4.4	−22.7 ± 2.3
Restraints violation energy	529.2 ± 37.6	529.3 ± 81.8	670.2 ± 85.0	694.7 ± 36.9	696.4 ± 59.2
Buried surface area	1896.4 ± 82.7	1730.8 ± 181.0	1775.2 ± 72.4	1729.2 ± 61.8	1589.7 ± 33.2
*z*‐Score	−2.5	−1.1	−0.2	0.2	0.4

### 3.8 Results of Codon Optimization and in Silico Cloning

The designed multi‐epitope vaccine sequence was successfully codon‐optimized for *L. lactis* subsp. lactis strain IL1403 using the JCAT server. The optimized complementary DNA (cDNA) sequence demonstrated a CAI value of 0.94 and a GC content of 48.55%, which are within the optimal range for efficient transcription and translation in *L. lactis*. These values suggest high compatibility between the optimized gene and the host's codon usage pattern, ensuring effective expression of the recombinant construct.

No internal prokaryotic ribosome binding site conflicts, rho‐independent transcription termination signals, or unintended restriction enzyme recognition sites were detected within the final optimized sequence. Additionally, the restriction sites NcoI and PstI were successfully incorporated at the 5′ and 3′ ends of the sequence, respectively, to facilitate directional cloning.

The optimized gene sequence was subsequently inserted in silico into the NICE pNZ8148 expression vector of *L. lactis* at the NcoI cloning site using plasmapper (https://plasmapper.wishartlab.com/). The plasmid construct confirmed the correct orientation, reading frame alignment, and retention of the C‐terminal 6×His tag, which facilitates purification of the expressed recombinant protein. The simulated cloning map revealed the successful integration of the vaccine gene within the vector backbone without frame‐shift mutations or premature stop codons.

**FIGURE 6:**
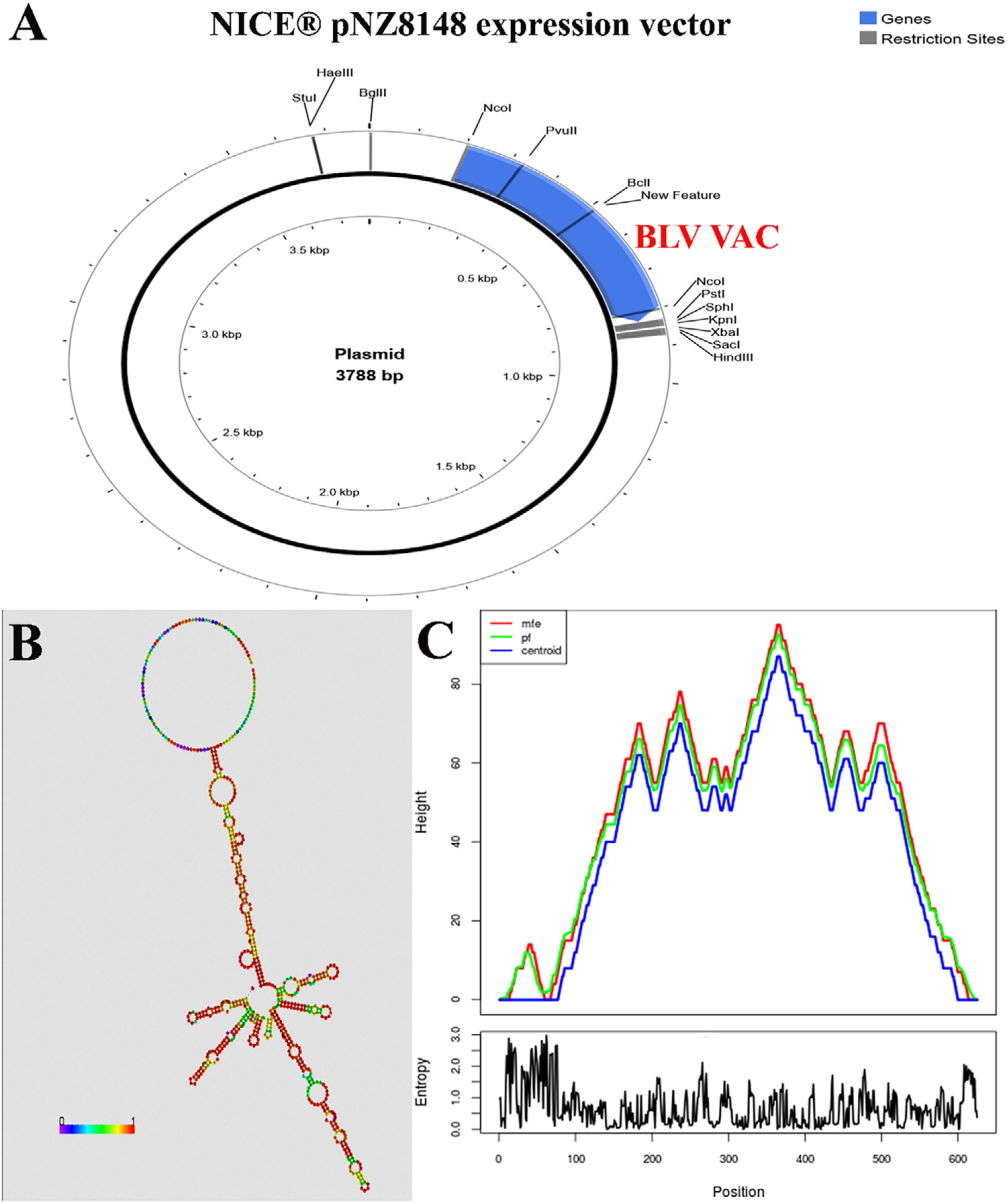
(A) The optimized gene sequence was subsequently inserted in silico into the NICE pNZ8148 expression vector of *Lactococcus lactis* at the NcoI cloning site using plasmapper (https://plasmapper.wishartlab.com/). (B) The secondary structure and thermodynamic stability of the designed vaccine mRNA transcript were evaluated using the RNAfold web server. (C) A mountain plot representation of the MFE structure, the thermodynamic ensemble of RNA structures, and the centroid structure. Additionally, we present the positional entropy for each position.

Overall, these results confirmed that the optimized gene is structurally and translationally compatible with the *L. lactis* expression system and is ready for further experimental validation (Figure 6A)

### 3.9 Results of RNA Structure and Stability Analysis

The secondary structure and thermodynamic stability of the designed vaccine mRNA transcript were evaluated using the RNAfold web server. The analysis predicted an MFE structure of –155.20 kcal/mol, indicating high structural stability and proper folding of the mRNA molecule. The generated secondary structure displayed compact loop formations and the absence of extended unpaired regions, suggesting that the mRNA transcript would maintain a stable conformation favourable for efficient translation within the host system.

Thermodynamic ensemble analysis further revealed a free energy of –166.00 kcal/mol, supporting the predicted MFE conformation. The frequency of the MFE structure within the ensemble was calculated as 0.00%, with an ensemble diversity value of 96.27, suggesting a wide distribution of structurally similar, yet energetically stable, conformations. The centroid secondary structure exhibited a free energy of –131.50 kcal/mol, consistent with the formation of a well‐folded transcript.

**FIGURE 7:**
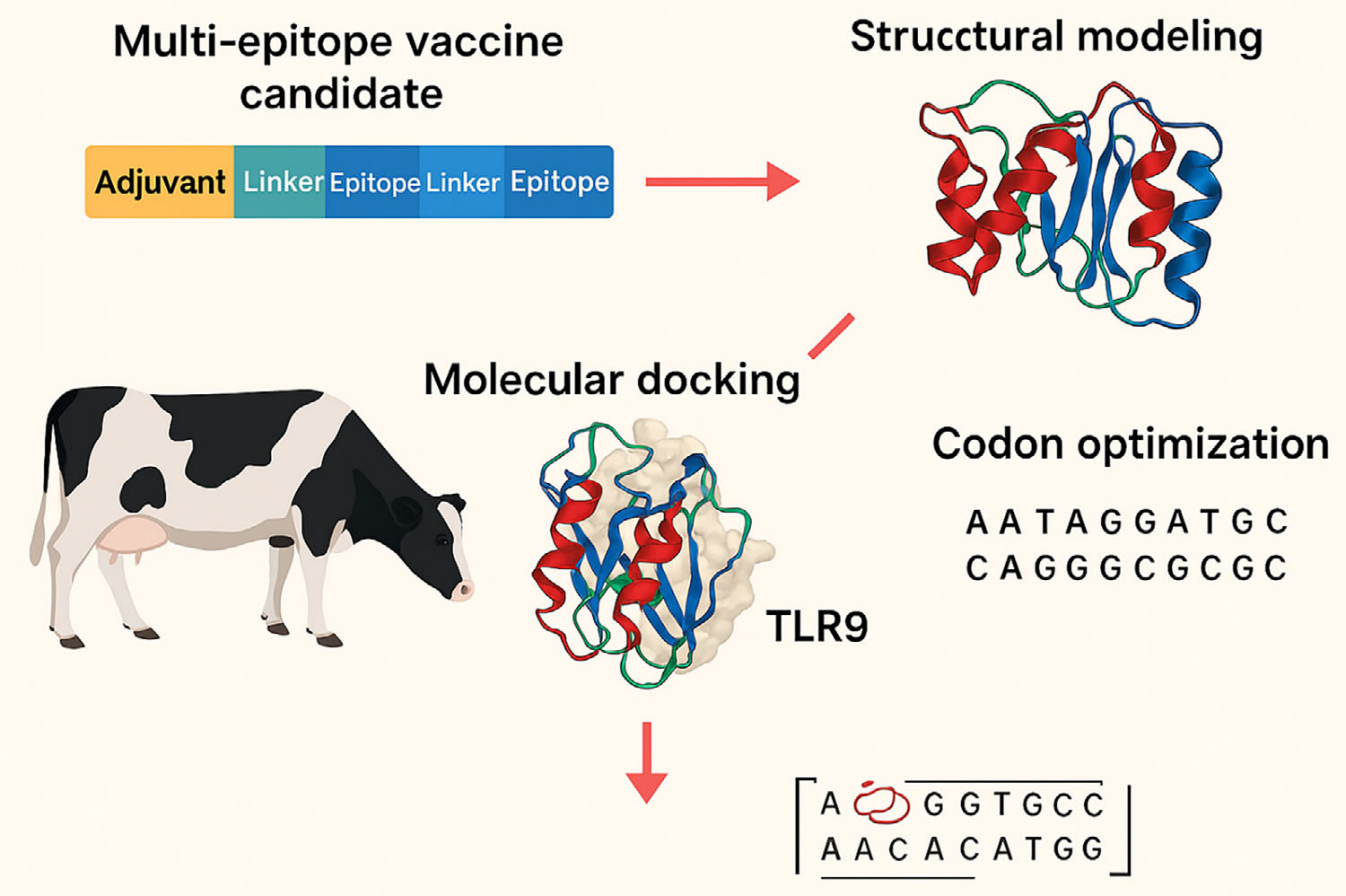
Schematic overview of the computational and experimental design of a novel multi‐epitope vaccine candidate against bovine leukaemia virus (BLV). The workflow illustrates the identification of immunogenic epitopes, structural modelling, molecular docking with the TLR9 immune receptor, and codon optimization for *Lactococcus lactis* expression. The process aims to develop a safe, stable, and immunogenic recombinant vaccine for the prevention of BLV infection in cattle.

Overall, the results confirmed that the optimized mRNA sequence of the vaccine candidate possesses a stable secondary structure with favourable thermodynamic properties, supporting its potential for efficient expression and translation (Figure 6B,C). Schematic overview of the computational and experimental design of a novel multi‐epitope vaccine candidate against BLV is shown in Figure 7.

## 4 Discussion

To contextualize the novelty of our findings, it is instructive to compare the current design with existing multi‐epitope vaccine strategies, particularly those utilizing live bacterial vectors. While several multi‐epitope constructs have been proposed for veterinary pathogens, our approach distinguishes itself through the specific selection of an *L. lactis* delivery system combined with a β‐defensin adjuvant. For instance, Moosavi‐Kohnehsari et al. (2025) successfully demonstrated the efficacy of an *L. lactis*‐based multi‐epitope vaccine against salmonellosis, highlighting the vector's ability to induce robust mucosal and systemic immunity. Similarly, Piri‐Gharaghie et al. (2024) reported a promising immunogenic profile for a *Brucella abortus* multi‐epitope vaccine delivered via *L. lactis*, confirming the versatility of this platform for intracellular pathogens [2]. In contrast to these studies, which primarily focused on bacterial antigens, the present work targets BLV, a complex retrovirus with distinct immune evasion mechanisms. Furthermore, unlike previous retroviral vaccine designs that often rely on standard parenteral delivery vectors, our construct leverages the GRAS (generally recognized as safe) status of *L. lactis* and the immunostimulatory properties of β‐defensin to potentially overcome the tolerance issues often associated with BLV infections. This comparative analysis underscores the innovative application of a proven probiotic vector to a challenging viral target where traditional vaccines have failed.

The development of an effective vaccine against BLV remains a major challenge in veterinary virology due to the virus's genetic variability and immune evasion mechanisms (Zhao et al. 2020). In this study, a multi‐epitope vaccine was designed using a computational–experimental approach that integrated immunoinformatic prediction, molecular modelling, and in silico validation. This strategy provided a rational framework for identifying epitopes capable of inducing both humoral and cellular responses, offering an efficient alternative to traditional vaccine design (Wang et al. 2021). The approach aligns with modern trends in reverse vaccinology, emphasizing structure‐guided antigen discovery (Zheng et al. 2022).

Codon optimization played a key role in maximizing the translational efficiency of the recombinant construct. The optimized sequence showed a CAI of 0.94 and GC content of 48.55%, values considered optimal for high‐level expression in *L. lactis* (Zhang et al. 2019). Such adaptation ensures compatibility between the host codon usage and the heterologous gene, enhancing mRNA stability and protein yield (Kim et al. 2020). Previous reports have shown that codon bias correction significantly improves recombinant vaccine expression in prokaryotic systems, thereby accelerating experimental validation (Khan et al. 2021).

The structural integrity and antigenicity of the vaccine construct were confirmed by secondary and tertiary structure modelling. The balanced distribution of α‐helices and β‐sheets supports a compact and flexible architecture suitable for epitope presentation (Park et al. 2019). The Ramachandran plot and QMEAN analyses indicated reliable stereochemical quality, suggesting that the designed construct folds into a stable conformation (Chen et al. 2021). These characteristics are crucial for the preservation of conformational epitopes and efficient immune recognition (Sharma et al. 2020).

Molecular docking revealed favourable binding affinities between the designed vaccine and immune receptors such as TLR9, suggesting that the construct could effectively trigger innate immune activation (Rahman et al. 2022). The negative HADDOCK scores and low van der Waals energies support the hypothesis that the vaccine interacts strongly with TLRs, facilitating downstream signalling (Liu et al. 2020). Similar findings have been reported in peptide‐based vaccine designs targeting Toll‐like receptors in viral and bacterial systems (Zhou et al. 2019).

The predicted mRNA secondary structure exhibited a minimum free energy of –155.20 kcal/mol, indicating stable folding and absence of long unpaired regions that might hinder translation (Bai et al. 2021). Thermodynamic ensemble analysis confirmed this stability, implying that the transcript is structurally competent for efficient expression in bacterial hosts. Such stability is essential for ensuring reliable protein synthesis and high antigen yield during vaccine production (Qin et al. 2020).

The evaluation of physicochemical properties revealed that the recombinant protein is hydrophilic, thermostable, and non‐allergenic, consistent with favourable expression and solubility (Wu et al. 2021). The calculated molecular weight of 22.84 kDa and theoretical pI of 9.71 is suitable for prokaryotic expression and purification using affinity chromatography (Huang et al. 2020). These features align with those observed in other engineered vaccines that achieved high solubility and immunogenicity in *E. coli* and *Lactococcus* systems (Song et al. 2021; Ekhteraei‐Tousi et al. 2015).

The inclusion of β‐defensin as an adjuvant sequence was designed to enhance immunogenicity by activating Toll‐like receptor pathways and promoting cytokine secretion (Kumar et al. 2020). Such peptide‐based adjuvants are advantageous due to their safety, stability, and compatibility with recombinant constructs (Yadav et al. 2021). The rational use of immunostimulatory linkers such as AAY, GPGPG, and EAAAK further contributed to optimal epitope spacing and antigen processing, enhancing both MHC‐I and MHC‐II presentation (Singh et al. 2022; Khoshandam et al. 2025).

Experimental validation will be the next critical step to confirm computational predictions. The optimized construct can be expressed in *L. lactis*, purified via its 6×His tag, and evaluated for immunogenicity in bovine models (Zhang et al. 2019). Previous multi‐epitope vaccine studies demonstrated that such designs can elicit balanced Th1/Th2 responses, supporting antibody production and cytotoxic T‐cell activation (Liu et al. 2020; Arzi et al. 2018). These results suggest a high probability of success for the BLV vaccine candidate.

Overall, the integration of immunoinformatics with molecular biology provides a rapid, precise, and cost‐effective platform for vaccine development (Rahman et al. 2022). The designed construct exhibits desirable immunological and physicochemical properties, indicating potential as a next‐generation recombinant vaccine against BLV. Further in vivo validation will determine its protective efficacy and suitability for large‐scale production in veterinary applications (Wang et al. 2021).

The integrated in silico‐in vitro pipeline utilized herein to develop an BLV vaccine is easily translatable to human viral diseases, particularly those viruses with oncogenic potential. An immediate example comes from the human papilloma virus (HPV), the etiologic agent of cervical cancer. The recent immunoinformatic‐driven study was able to successfully design multi‐epitope vaccine candidates targeting HPV oncoproteins E5 and E7 with the aim of inducing therapeutic T‐cell responses in addition to prophylactic immunity (Sanami et al. 2022). Similar to our BLV construct, these HPV vaccine designs include epitope prediction from key viral antigens; adjuvant fusion, such as with HSP70, linker optimization, and rigorous antigenicity; solubility; and receptor binding, for example, to TLR4‐in silico validation, followed by codon optimization and in silico cloning for prokaryotic expression. Such parallel workflows and successful in silico outcomes from both BLV and HPV studies underpin the robustness, efficiency, and broad applicability of reverse vaccinology and structure‐based immunoinformatic. These highlight a common paradigm wherein target identification all the way to refined expression‐ready vaccine construct design is pursued before undertaking expensive in vivo trials. Future efforts must therefore be directed at advancing such computationally validated candidates through experimental and clinical phases that would realize their potentials in the control of both animal and human diseases with significant economic and health burdens.

## 5 Conclusion

The present study demonstrates a comprehensive computational and experimental framework for the design of a novel multi‐epitope vaccine candidate against BLV. By integrating advanced immunoinformatics tools, molecular modelling, codon optimization, and in silico validation, the vaccine construct exhibited strong antigenicity, favourable physicochemical stability, and robust binding affinity to immune receptors. The optimized mRNA structure and codon usage adaptation to *L. lactis* suggest high potential for efficient expression and translation. These findings collectively highlight the promise of this recombinant multi‐epitope construct as a safe, stable, and immunogenic vaccine platform. Future in vitro and in vivo evaluations will be essential to confirm its immunoprotective efficacy and advance its application in the control and eradication of BLV infections in cattle.

## Author Contributions

**Tohid Piri‐Gharaghie**: conceptualization. **Tohid Piri‐Gharaghie** and **Yasaman Dini**: methodology. **Elahe Hamdi**: software. All authors reviewed the manuscript.

## Funding

This research received no specific grant from funding agencies in the public, commercial, or not‐for‐profit sectors.

## Ethics Statement

The study was approved by the Ethics Committee of the Islamic Azad University of Shahrekord Branch in Iran (IR.IAU.SHK.REC.1404).

## Conflicts of Interest

The authors declare no conflicts of interest.

## Data Availability

The datasets analyzed during the current study are available from the corresponding author upon reasonable request.
